# Visualizing Thermal Reduction in Graphene Oxide

**DOI:** 10.3390/ma18102222

**Published:** 2025-05-11

**Authors:** Xiangrui Xu, Junjie Huang, Gesong Miao, Bo Yan, Yangbo Chen, Yinghui Zhou, Yufeng Zhang, Xueao Zhang, Weiwei Cai

**Affiliations:** 1 College of Physical Science and Technology, Xiamen University, Xiamen 361005, China; 2 Jiujiang Research Institute of Xiamen University, Jiujiang 360404, China

**Keywords:** graphene oxide, reduction, optical microscopy, X-ray photoelectron spectroscopy, Raman spectroscopy

## Abstract

The reduction of graphene oxide (GO) is critical for tuning its properties. This study integrates optical contrast analysis with Raman spectroscopy and X-ray photoelectron spectroscopy (XPS) to investigate the structural and optical evolution of GO in thermal reduction. For GO on 100 nm SiO_2_/Si, the R channel contrast exhibits superior sensitivity to structural changes, making it a reliable indicator of the reduction process. A theoretical model based on Fresnel equations reveals the role of SiO_2_ thickness in modulating optical contrast, providing guidelines for substrate optimization and channel selection.

## 1. Introduction

Due to its unique layered structure with oxygen-containing functional groups, GO has become a significant functional material for various applications, such as photonics [1,2,3,4], electronic [5,6,7], sensors [8,9], and energy technologies [10,11]. Thermal reduction is commonly used to remove oxygen-containing functional groups from GO and restore the sp^2^-conjugated carbon framework. The reduction parameters, such as temperature and duration, exert a substantial influence on the reduction effect, thereby modulating the resulting properties [12,13,14,15,16]. Understanding reduction processes is crucial for optimizing the application potential of GO. Hence, various techniques have been employed to investigate the reduction [12,13,14,15,16,17,18,19,20,21].

Raman spectroscopy is commonly used to investigate the structural change in graphene-based material, with a focus on the characteristic features (i.e., the D and G modes). The D mode arises from out-of-plane vibrations induced by structural defects and disorders in the carbon lattice, including oxygenated functional groups, as well as double-resonant processes near the Brillouin Zone boundary. Meanwhile, the G mode originates from in-plane vibrations of the sp^2^-hybridized carbon framework, corresponding to the first-order scattering of the E_2g_ mode [17,18,22,23,24,25]. Hence, the intensity ratio of these modes (I_D_/I_G_) and the full width at half maximum (FWHM) are widely deployed to reveal the structural changes during the GO reduction process [17,26,27,28,29,30]. On the other hand, XPS is employed to understand the electronic and chemical structure of materials. Hence, the GO reduction progress can be analyzed by XPS (e.g., the change in oxygen-containing functional groups). However, inconsistencies in peak selection and binding energy determination during spectral deconvolution have been widely reported [18,21,31,32,33]. The C 1s peak shift observed in GO relative to pristine graphene complicates charge correction procedures [32]. Moreover, the uncertainty of fitting parameters, especially for the asymmetric C-C sp^2^ signal, affects the reliability of quantitative analysis [21]. While Raman spectroscopy is a powerful tool, it may require careful optimization of parameters (e.g., laser intensity, exposure time) to minimize sample damage and improve efficiency, especially for large-scale measurements.

In contrast, despite its inability to directly provide specific structural information, optical microscopy offers a rapid, cost-effective, and non-destructive approach for analyzing properties like thickness and oxidation degree in two-dimensional materials, including graphene [34,35,36,37], transition metal dichalcogenides [38,39,40,41,42,43,44], and others [45,46,47,48,49,50]. Changes in optical images and contrast changes extracted from them are used to evaluate the oxidation process of WTe_2_ [51]. Changes in optical images are used to evaluate the oxidation process of MoTe_2_ and MoTe_2_ encapsulated by MoS_2_ to evaluate the protective effect of MoS_2_ [52]. Changes in optical images and contrast changes extracted from them are used to evaluate the oxidation process of MoTe_2_ and MoTe_2_ encapsulated by hexagonal boron nitride to evaluate the protective effect of hexagonal boron nitride [53]. Optical microscopy is used to identify the thickness of GO processed by vacuum heating to improve the optical contrast [54] and estimate the reduction degree of GO by correlating of XPS by optical contrast [27]. However, the evolution of optical contrast in various wavelength region during GO reduction and its physical mechanisms have not been comprehensively investigated.

In this work, an approach combining multi-channel optical contrast analysis with Raman spectroscopy and XPS is proposed to investigate the GO reduction process. It is found that optical contrast allows rapid assessment of reduction processes, while Raman spectroscopy and XPS provide detailed structural information. Different channels of optical contrast have different sensitivities to thermal reduction. For GO on 100 nm SiO_2_/Si, the R channel contrast in optical images exhibits superior sensitivity to thermal reduction. A theoretical model based on Fresnel equations reveals the evolution of optical properties across distinct color channels and the modulation of these properties by SiO_2_ layer thickness, providing guidelines for channel selection and substrate optimization for contrast analysis.

## 2. Method

A GO aqueous suspension (1.5 mg/mL) is prepared using a modified Hummers method [55] and spin-coated onto 100 nm SiO_2_/Si substrates pre-treated with oxygen plasma (Harrick Plasma, PDC-32G-2, Ithaca, New York, NY, USA) for 10 min. The spin-coating process consists of two steps: 1500 rpm for 30 s and 3000 rpm for 60 s, ensuring uniform GO deposition. GO reduction is performed on 100 nm SiO_2_/Si substrates in a tube furnace at temperatures of 200, 400, 600, and 800 °C under a vacuum of less than 1 Pa for 30 min without gas.

Optical images are acquired using a ZYJ-1000E optical microscope(China, Shanghai). Python code utilizing multiple libraries is employed to generate individual channel images and measure intensities. For individual channel image generation, Pillow (10.4.0) (PIL) is employed for RGB image reading and channel separation, generating pseudo-color images. For contrast quantification, OpenCV (4.10.0) (cv2) is used for image reading with interactive ROI selection to annotate substrate and material regions, NumPy (1.26.4) for calculating grayscale and R, G, and B channel contrasts. Raman measurements are performed at room temperature using a WITec Alpha-300 (Germany, Baden-Württemberg, Ulm) with a blue laser of 488 nm for stronger Raman signals. XPS measurements are performed using a Thermo Scientific K-Alpha (Waltham, MA, USA) with Al Kα radiation (1486.6 eV) as the excitation source. Chamber vacuum less than 2.0 × 10^−7^ mBar. The resolution of XPS spectra used is 0.1 eV. Atomic force microscopy (AFM) is performed using a NTEGRA Prima (Russia, Moscow).

## 3. Results and Discussion

**Figure 1:**
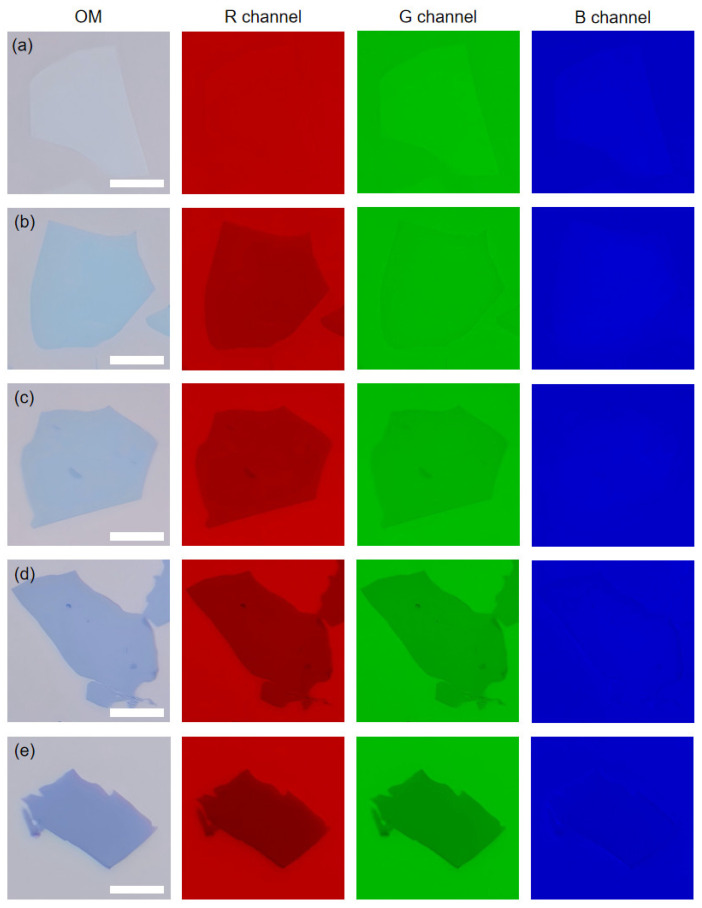
Optical and channel-separated images of GO before reduction (**a**) and after reduction at 200 °C (**b**), 400 °C (**c**), 600 °C (**d**), and 800 °C (**e**). The scale bars are 10 μm.

Figure 1 presents optical and channel-separated images of GO before and after thermal reduction at different temperatures. The first column are optical mages, and the next three columns are pseudo-color images of the R, G, and B channels, respectively. While the visibility of GO on a 100 nm SiO_2_/Si substrate initially appears poor, it progressively improves with increasing reduction temperature, consistent with previous reports [27,54]. However, distinct phenomena emerge in channel-separated images. R channel images show near-indistinguishable GO initially, followed by rapid visibility enhancement during reduction, surpassing optical image visibility. In G channel images, GO is always distinguishable and also shows a rapid increase in visibility. However, the visibility is poorer than that in R channel images. Although visibility in B channel images also increases with temperature, it remains significantly lower than in other channels. This channel-dependent visibility variation reveals wavelength-specific optical responses during GO reduction and offers an optical method for monitoring graphene oxide reduction.

To complement the optical microscopy findings and provide a comprehensive understanding of the reduction process, Raman spectroscopy and XPS are applied for detailed chemical and structural characterization. These techniques enable examination of oxygen-containing functional group removal and sp^2^-hybridized carbon framework restoration, offering critical insights into the reduction mechanism.

**Figure 2:**
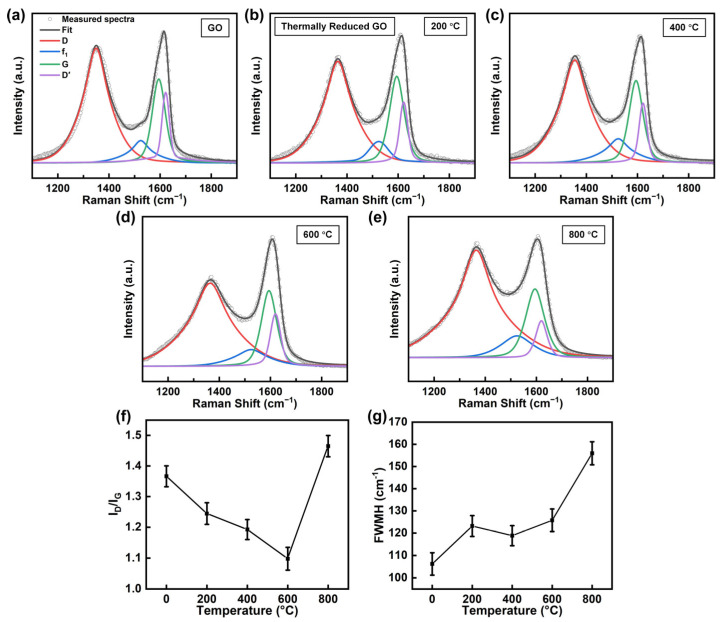
(**a**–**e**) Raman spectra in the 1000–2000 cm^−1^ region with deconvoluted peaks, (**f**) I_D_/I_G_ ratio, and (**g**) FWHM of G mode for GO before and after reduction at various temperatures.

Figure 2a–e show the representative Raman spectra in the 1000–2000 cm^−1^ region with deconvoluted peaks of GO deposited on 100 nm SiO_2_/Si substrates before and after reduction at various temperatures. Following spectral calibration with the silicon reference peak (520 cm^−1^) and baseline subtraction using Asymmetric Least Squares Smoothing, peaks are carefully fitted with a Voigt function, which accounts for both Gaussian and Lorentzian contributions. The fitting process employs the Levenberg-Marquardt algorithm for optimization. The G and D′ peaks overlap, while the D, G, and D′ peaks alone are insufficient to accurately fit all Raman signals. Therefore, an additional f_1_ peak located between the D and G peaks is included to improve the fitting results [56,57]. The complete Raman spectra are shown in Figure S1. The spectra exhibit characteristic D and G modes at ~1330 cm^−1^ and ~1600 cm^−1^, respectively [58]. The intensity ratio of these modes (I_D_/I_G_) provides information on the defects [28,29,59], thereby accessing the structural changes during the reduction process. The I_D_/I_G_ ratio decreases with increasing annealing temperature up to 600 °C, then rises with further increasing temperature, as depicted in Figure 2f. The initial decrease in the I_D_/I_G_ ratio suggests the removal of oxygen-containing functional groups and the recovery of sp^2^-hybridized carbon framework, accompanied by a reduction in defects, resulting in a corresponding decrease in the intensity of the D mode relative to the G mode. However, at 800 °C, the increase in the I_D_/I_G_ ratio suggests the emergence of new structural defects.

Figure 2g shows the temperature-dependent variation in the G mode FWHM of GO. The initial G mode FWHM of 106.2 cm^−1^ increases to 123.2 cm^−1^ at 200 °C, remains stable at 120.1 cm^−1^, and 125.2 cm^−1^ for 400 °C and 600 °C respectively, and shows a significant increase to 156.1 cm^−1^ at 800 °C. The observed G mode broadening at 200 °C suggests a decrease in the crystalline quality of the sp^2^-hybridized carbon framework. The nearly invariant FWHM values between 400–600 °C indicate that the defect density of the sp^2^-hybridized carbon framework remains stable during intermediate thermal treatment. The FWHM increases at 800 °C, indicating another decrease in crystalline quality. This phenomenon is likely attributed to the formation of Stone-Wales defects, vacancies, and distortions during high-temperature annealing, leading to G mode broadening [60,61,62,63]. The non-monotonic behavior of the I_D_/I_G_ ratio and G mode FWHM complicates the detection of the reduction of GO.

**Figure 3:**
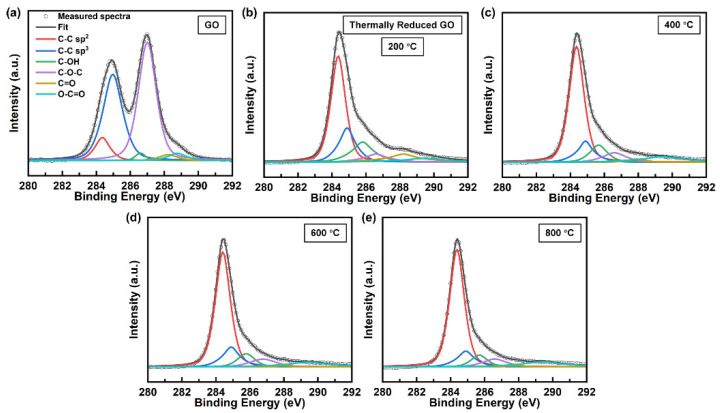
C 1s XPS spectra with deconvoluted peaks of GO before reduction (**a**) and after reduction at 200 °C (**b**), 400 °C (**c**), 600 °C (**d**), and 800 °C (**e**).

Figure 3 shows the representative C 1s XPS spectra of GO before and after reduction at various temperatures. The C 1s XPS spectra are deconvoluted using six Voigt functions for characteristic peaks after Shirley background subtraction [64]: C-C sp^2^ (284.4 eV), C-C sp^3^ (285 eV), C-O (285.7 eV), C-O-C (286.7 eV), C=O (288.0 eV), and O-C=O (290.1 eV) [21,64,65]. The mathematical reliability was assessed using the coefficient of determination, analysis of variance, and residual analysis to ensure statistically sound fitting results. The content of C 1S chemical groups is shown in Table S1. Thermal annealing progressively restores the graphitic structure in GO, as evidenced by the significant increase in C-C sp^2^ content from 16% to 69% and the corresponding decrease in C-C sp^3^ content from 24% to 10% after annealing at 800 °C. This transformation indicates effective recovery of the sp^2^-hybridized carbon framework and defect healing. The dominant oxygen-containing functional C-O-C group decomposed rapidly at 200 °C and stabilized at higher temperatures, while the percentage of minor oxygen-containing functional groups (C-OH, C=O, and O-C=O) is consistently lower. XPS analysis effectively tracks chemical changes during the initial reduction stage (GO to 200 °C), where C-C sp^2^ content increased sharply from 16% to 48% and sp^3^ content decreased from 24% to 17%. However, its sensitivity diminished for detecting further structural changes during advanced thermal treatments (600 °C to 800 °C), as evidenced by the increase in C-C sp^2^ content (63% to 69%) and decrease in C-C sp^3^ content (12% to 10%). While they can still be distinguished, the discrepancy in C-C sp^2^ content and C-C sp^3^ content between the two temperatures is reduced.

Clearly, the change in optical images of GO closely correlates with the reduction temperature as well as the structural change in GO. To quantitatively analyze the change, the contrast (*C*) of the GO flake is defined as:

(1) $$C=\frac{I_{substrate}-I_{flake}}{I_{substrate}}.$$

where $I_{substrate}$ represents the substrate intensity and $I_{flake}$ represents the GO flake intensity. A code programmed in Python (3.12.3) is employed for measuring intensities, as described in Method. This measure provides a numerical representation of the visual distinction between the GO and the underlying 100 nm SiO_2_/Si substrate. Note that a higher contrast value indicates a more distinct boundary between the GO and the substrate.

**Figure 4:**
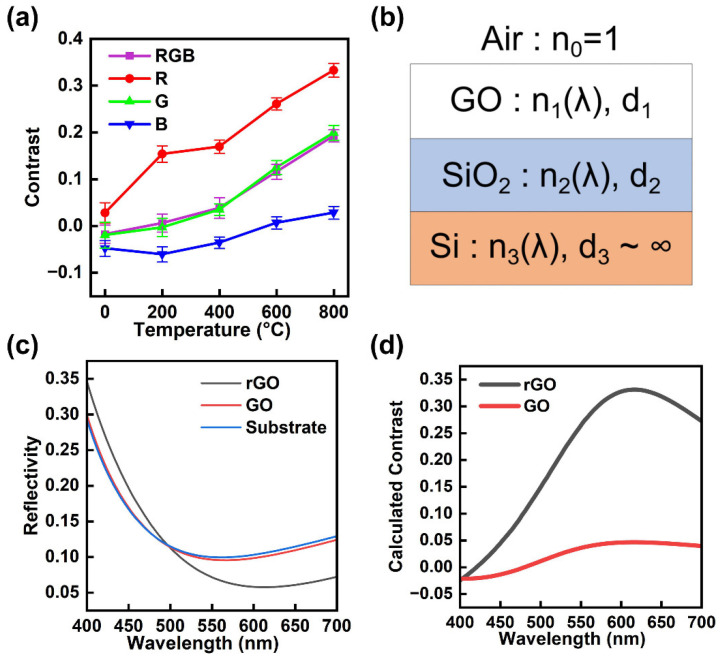
(**a**) Contrast of optical images (RGB) and channel-separated images before and after reduction at 200 °C, 400 °C, 600 °C, and 800 °C. (**b**) Schematic diagram of the GO/SiO_2_/Si structure. (**c**) Calculated reflectivity spectra of rGO, GO, and the 100 nm SiO_2_/Si substrate. (**d**) Calculated contrast spectra of rGO and GO.

Figure 4a demonstrates the statistical contrasts of optical images (RGB) and their channel-separated counterparts, illustrating a notable enhancement in contrast throughout the reduction process. The data points are the mean values obtained from measurements of three GO flakes in independent experiments conducted under the same conditions, and the error bars indicate the standard deviation. At different thermal reduction temperatures, the contrast of the different channels of GO changes differently, and this stems from the structural changes induced by the reduction process. The pristine GO exhibits poor visibility, as evidenced by its negative RGB contrast at −0.01. Specifically, the B and G channels show negative contrasts of −0.04 and −0.02, respectively, and a negative contrast means that the color of the GO is lighter than the substrate. While only the R channel displays a positive contrast of 0.02. The contrast of the B channel suggests that pristine GO exhibits smaller contrasts in the blue wavelength range, which will be confirmed in the following theoretical analysis. As the reduction temperature increases to 200 °C, the R channel contrast rises at 0.15, while the B channel remains negative at −0.06. The G channel contrast approaches 0. The RGB contrast becomes positive at 0.01. As the reduction temperature increases to 400 °C, the R channel contrast increases at 0.17, and the G channel increases at 0.03. The B channel contrast weakens at −0.03, which still suggests smaller contrasts in the blue wavelength range. The RGB contrast was enhanced at 0.04. As reduction temperatures rise above 600 °C, the optical properties continue to change. In the R channel, contrast surges from 0.26 at 600 °C to 0.33 at 800 °C. This increase is accompanied by increases in both G (from 0.12 to 0.20) and B (from 0.01 to 0.03) channels. The RGB contrast increases from 0.12 to 0.19. Note that the B channel contrast transitions from negative to positive values from 400 °C to 600 °C.

These results highlight the significant differences in contrast enhancement between color channels and reveals that the R channel exhibits higher contrast, suggesting superior sensitivity to structural changes in GO during reduction, making the R channel contrast the preferred optical indicator of the reduction progress of GO.

The enhanced contrast resulting from thermal reduction is primarily attributed to the increased refractive index [54,66]. A model is developed to investigate the optical properties of GO (and GO after reduction), analyzing the interaction of normal-incidence light with a triple-layer structure (GO/SiO_2_/Si) as illustrated in Figure 4b. The optical contrast analysis is based on the Fresnel equations, which describe the reflection and transmission of light at the interface between two media with distinct refractive indices. The calculation requires the determination of the thickness *d*, and the refractive index *n(λ*), of each layer. Note that the thickness of the Si layer *d_3_*, is regarded as infinite. The thickness of the SiO_2_ layer *d_2_* is 100 nm. The thickness of GO *d_1_* is 0.8 nm, as demonstrated by AFM analysis, as shown in Figure S2. The reflection indexes are derived and optimized from the relevant literature [54,66,67,68,69,70].

As described in our previous work [71], the intensity of reflected light for normal incidence can be expressed as:

(2) $$I=p_{1}^{2}=|\frac{r_{1}e^{i(\delta_{1}+\delta_{2})}+r_{2}e^{-i(\delta_{1}-\delta_{2})}+r_{3}e^{-i(\delta_{1}+\delta_{2})}+r_{1}r_{2}r_{3}e^{i(\delta_{1}-\delta_{2})}}{e^{i(\delta_{1}+\delta_{2})}+r_{1}r_{2}e^{-i(\delta_{1}-\delta_{2})}+r_{1}r_{3}e^{-i(\delta_{1}+\delta_{2})}+r_{2}r_{3}e^{i(\delta_{1}-\delta_{2})}}{|}^{2},$$

where

(3) $$r_{1}=\frac{n_{0}-n_{1}}{n_{0}+n_{1}},r_{2}=\frac{n_{1}-n_{2}}{n_{1}+n_{2}},r_{3}=\frac{n_{2}-n_{3}}{n_{2}+n_{3}},$$

are the reflection coefficients at each interface determined by the Fresnel formula, while

(4) $$\delta_{1}=\frac{2\pi n_{1}d_{1}}{\lambda };\delta_{2}=\frac{2\pi n_{2}d_{2}}{\lambda },$$

are the phase thickness.

The reflection of the entire structure (*p*_1_), is calculated by the recursive method beginning with the bottom layer:

(5) $$p_{k}e^{i\varphi_{k}}=\frac{r_{k}+r_{k+1}e^{-2i\delta_{k}}}{1+r_{k}r_{k+1}e^{-2i\delta_{k}}};p_{k-1}e^{i\varphi_{k-1}}=\frac{r_{k-1}+p_{k}e^{i\varphi_{k}}e^{-2i\delta_{k-1}}}{1+r_{k-1}p_{k}e^{i\varphi_{k}}e^{-2i\varphi_{k-1}}};\ldots ;p_{1}.$$

Figure 4c shows the reflectivity spectra of GO, GO after reduction at 800 °C (rGO), and the 100 nm SiO_2_/Si substrate. The R, G, and B channels are defined as light in the 580–700, 480–600, and 400–500 nm ranges, respectively. The enhanced reflectivity in the B channel, accompanied by low overall reflectivity, produces a grey-blue tint in optical images. The reflectivity difference is significant in the R channel. The calculated contrast spectra of GO and rGO on 100 nm SiO_2_/Si are shown in Figure 4d, enabling quantitative analysis across different channels. The calculated contrast differences in RGB, R, G, and B channels are 0.19, 0.27, 0.22, and 0.007. These distinct contrast characteristics across wavelength ranges account for the differential channel responses observed in optical images and the superiority of the R channel.

**Figure 5:**
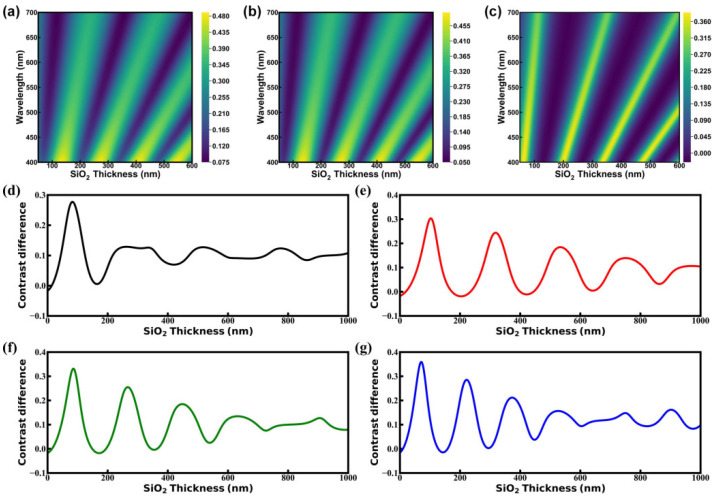
Contour plot of calculated reflectivity versus wavelength and SiO_2_ thickness of rGO (**a**) and GO (**b**) on 100 nm SiO_2_/Si substrate. (**c**) Contour plot of calculated contrast differences versus wavelength and SiO_2_ thickness. Calculated contrast differences versus SiO_2_ thickness in the RGB (**d**), R (**e**), G (**f**), and B (**g**) channels.

Note that the optical properties are sensitive to SiO_2_ thickness variations. Figure 5a,b show the calculated reflectivity contour plots of the GO and rGO on the 100 nm SiO_2_/Si substrate, exhibiting similar reflectance variation patterns with increasing SiO_2_ thickness, characterized by periodic intensity variations manifested as alternating light–dark bands with linear slopes and high reflectivity, which mostly occur in the short wavelength range, characterized by deep yellow in the lower part. Figure 5c shows the corresponding contrast difference contour plot, which demonstrates analogous periodic intensity modulations. SiO_2_ thickness variations induce distinct optical properties across color channels. While the R channel demonstrates the greatest contrast difference on 100 nm SiO_2_/Si substrates, specific SiO_2_ thickness ranges favor RGB, B, or G channels, characterized by yellow lines cutting across the plot. As demonstrated in Figure 5d–g, all channels exhibit periodic oscillatory behavior in contrast difference with SiO_2_ thickness, showing distinct periodicities and extremum values. The RGB channel demonstrates the shortest oscillation period, followed by the B, G, and R channels, as calculated by the difference between the wavelengths of the last and first extreme point divided by the number of extreme points. The ratio of the last extreme point to the first extreme point indicates that the R channel undergoes the most significant decay in contrast, followed by the G, RGB, and B channels.

## 4. Conclusions

In summary, an integrated approach combining optical contrast analysis with Raman spectroscopy and XPS is presented to investigate the reduction of GO. The capability of optical microscopy as a rapid and effective tool for characterizing the reduction process of GO on 100 nm SiO_2_/Si is demonstrated. The results show that the R channel contrast for this sample is highly sensitive to structural changes during thermal reduction, making it a reliable indicator of reduction progress. While Raman spectroscopy and XPS provide detailed structural insights, the observed non-monotonic behavior in Raman spectra and weakened but still effective discrimination of XPS data at high temperatures demonstrate the utility of optical contrast analysis for fast, large-area assessment of reduction progress. The theoretical model based on Fresnel equations provides insights into differences across color channels and how SiO_2_ layer thickness modulates optical properties, offering guidelines for substrate optimization and channel selection. In industrial settings, the selection of color channels sensitive to thermal reduction based on substrate materials enables rapid, non-destructive quality control. This approach proves particularly valuable for efficient material monitoring while preserving product integrity. Future research directions should focus on extending this methodology to other 2D materials like transition metal dichalcogenides to streamline post-synthesis characterization. Additionally, integrating automated optical contrast analysis with machine learning could significantly improve throughput and accuracy for industrial-scale material screening.

## Data Availability

All data that support the findings of this study are included within the article (and any Supplementary Files).

## Supplementary Materials

The following supporting information can be downloaded at: https://www.mdpi.com/article/10.3390/ma18102222/s1, Figure S1: Raman spectra of GO before and after reduction at various temperatures; Figure S2: AFM height image of GO on 100 nm SiO_2_/Si. The scale bar is 10 µm; Table S1: Content of C chemical groups.
