# Supplementary material for: Visualizing Thermal Reduction in Graphene Oxide
Source: Materials (Basel). 2025 May 11;18(10):2222. doi: 10.3390/ma18102222 (PMC12113108; doi:10.3390/ma18102222)
Supplement: Supplementary file 1 [file materials-18-02222-s001.zip › materials-3589242-supplementary.pdf]

Visualizing thermal reduction in graphene oxide

Xiangrui Xu[1], Junjie Huang[1], Gesong Miao[1], Bo Yan[1], Yangbo chen[1], Yinghui Zhou[1], Yufeng Zhang[1,2], Xue-ao Zhang[1,2*] and Weiwei Cai[1,2*]

1 College of Physical Science and Technology, Xiamen University, Xiamen 361005, People's Republic of China

2 Jiujiang research institute of Xiamen University, Jiujiang 360404, People's Republic of China

*Author to whom any correspondence should be addressed.

Supporting information

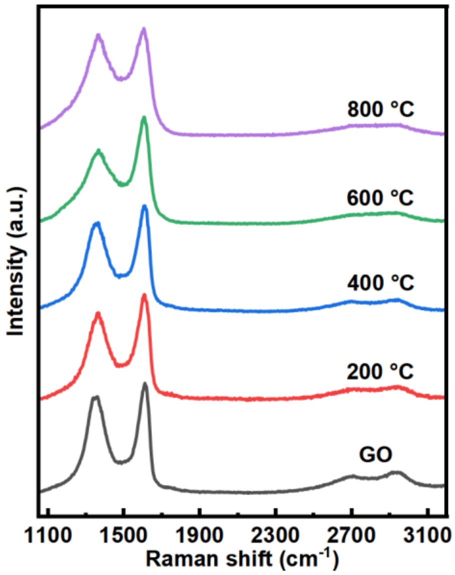

Figure S1 Raman spectra of GO before and after reduction at various temperatures.

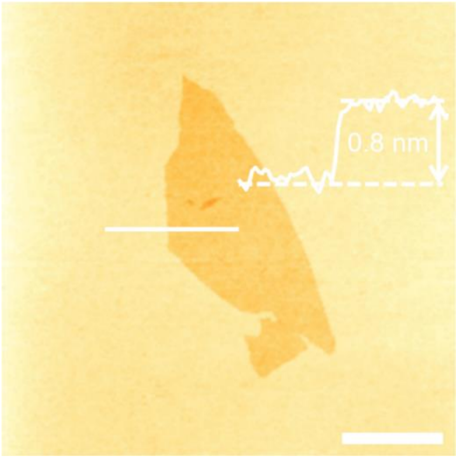

Figure S2 AFM height image of GO on 100 nm SiO_2/Si. The scale bar is 10 μm.

Table S1 Content of C chemical groups

Sample	C 1s group content (at%)			
	C-C sp^2	C-C sp^3	C-O-C	others
GO	16	24	44	16
200 ℃	48	17	7	28
400 ℃	60	12	7	21
600 ℃	63	12	7	18
800 ℃	69	10	7	14
